# Supplementary figures and images for: Multiple T6SSs, Mobile Auxiliary Modules, and Effectors Revealed in a Systematic Analysis of the Vibrio parahaemolyticus Pan-Genome
Source: mSystems. 2022 Oct 13;7(6):e00723-22. doi: 10.1128/msystems.00723-22 (PMC9765294; doi:10.1128/msystems.00723-22)

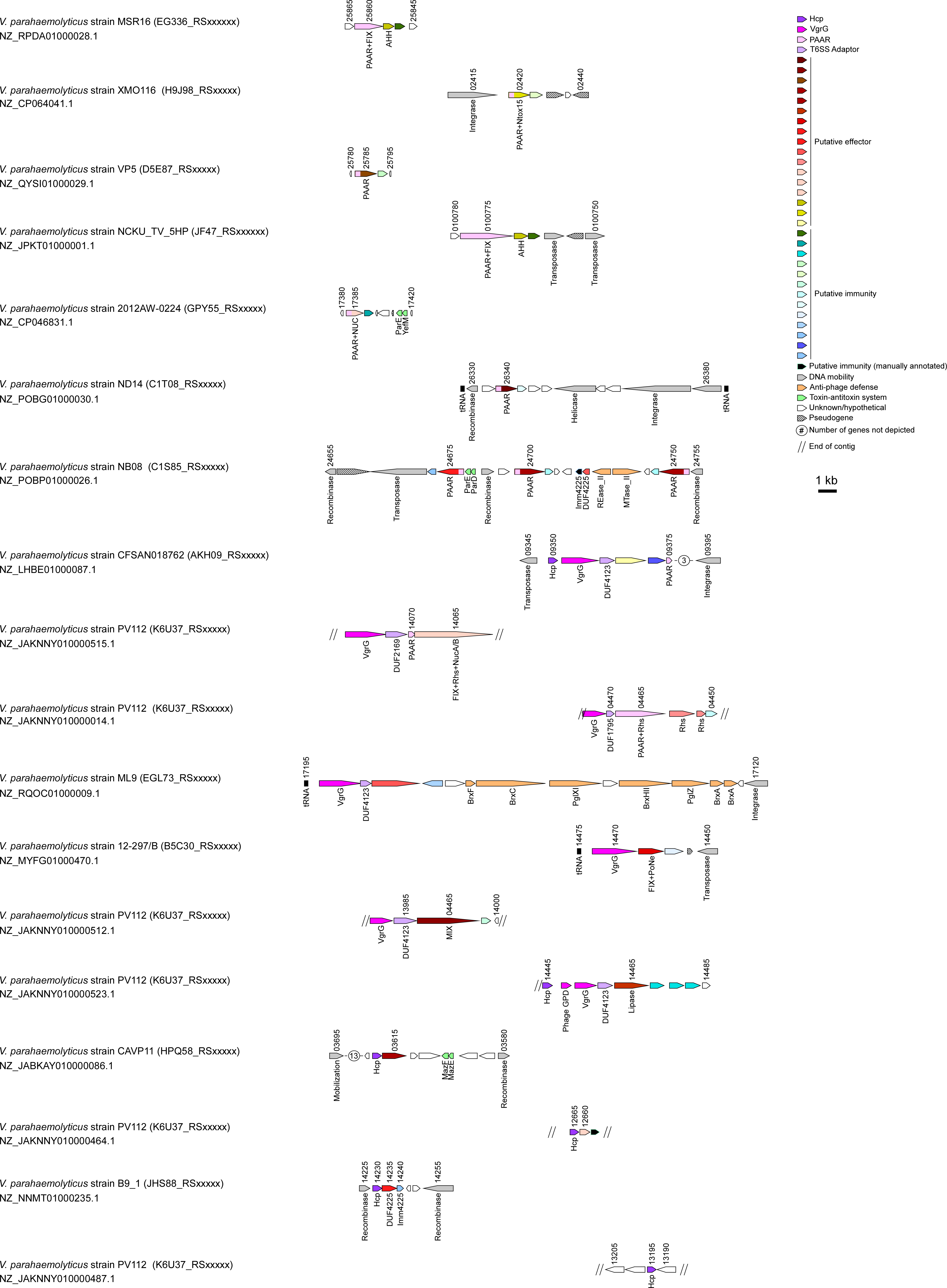

Supplement: FIG S1 [file msystems.00723-22-s0001.tif]

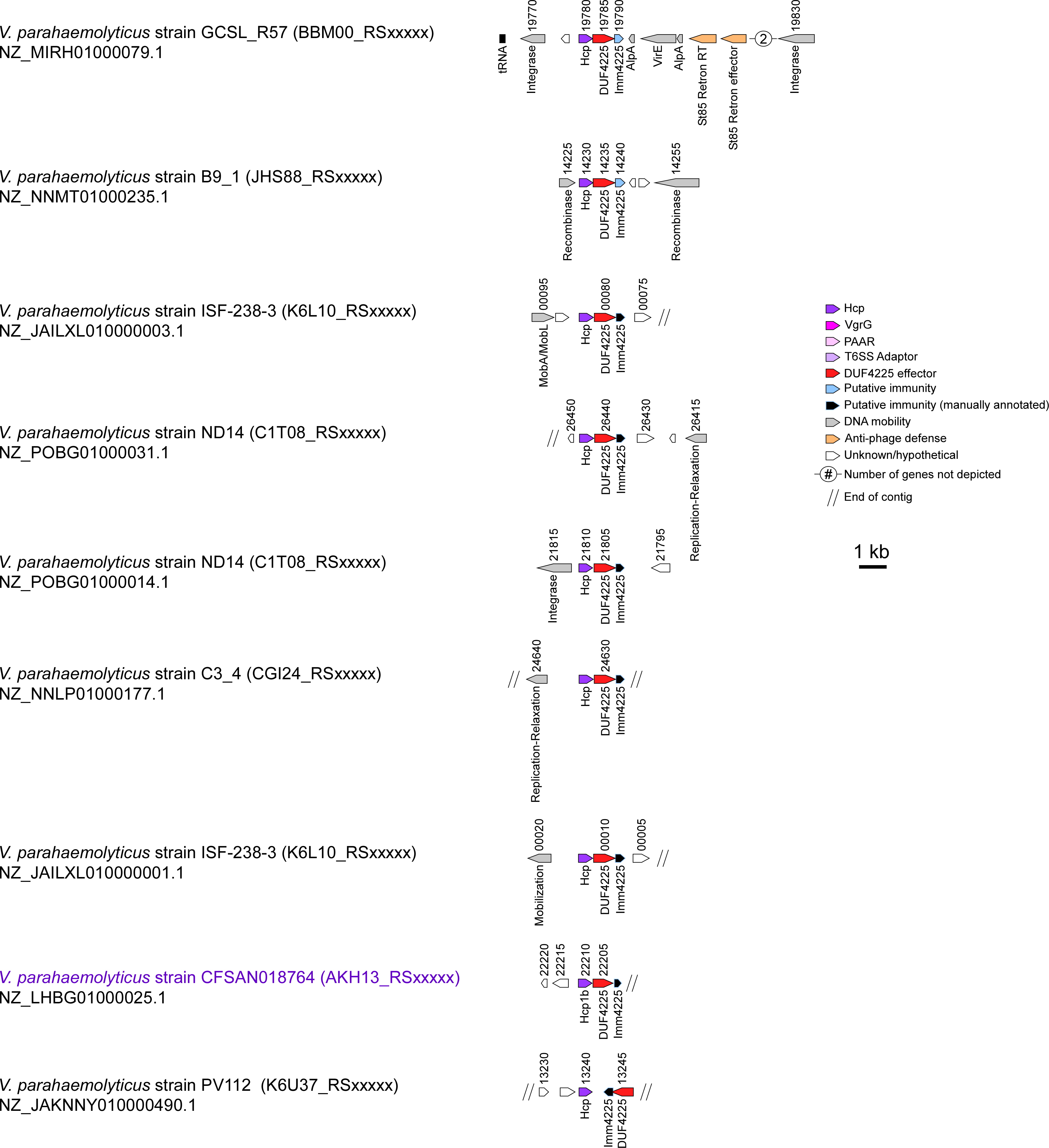

Supplement: FIG S2 [file msystems.00723-22-s0002.tif]

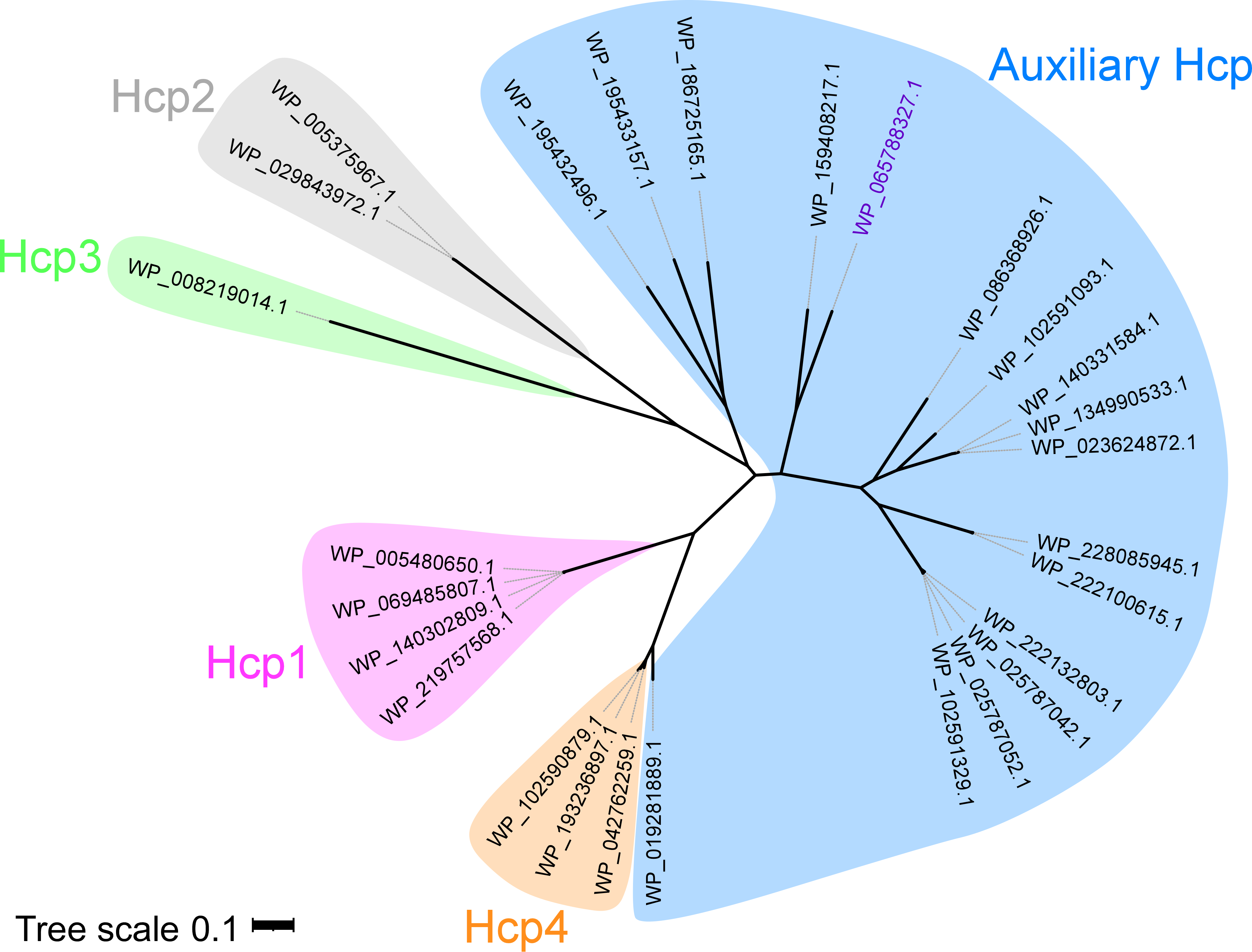

Supplement: FIG S3 [file msystems.00723-22-s0003.tif]

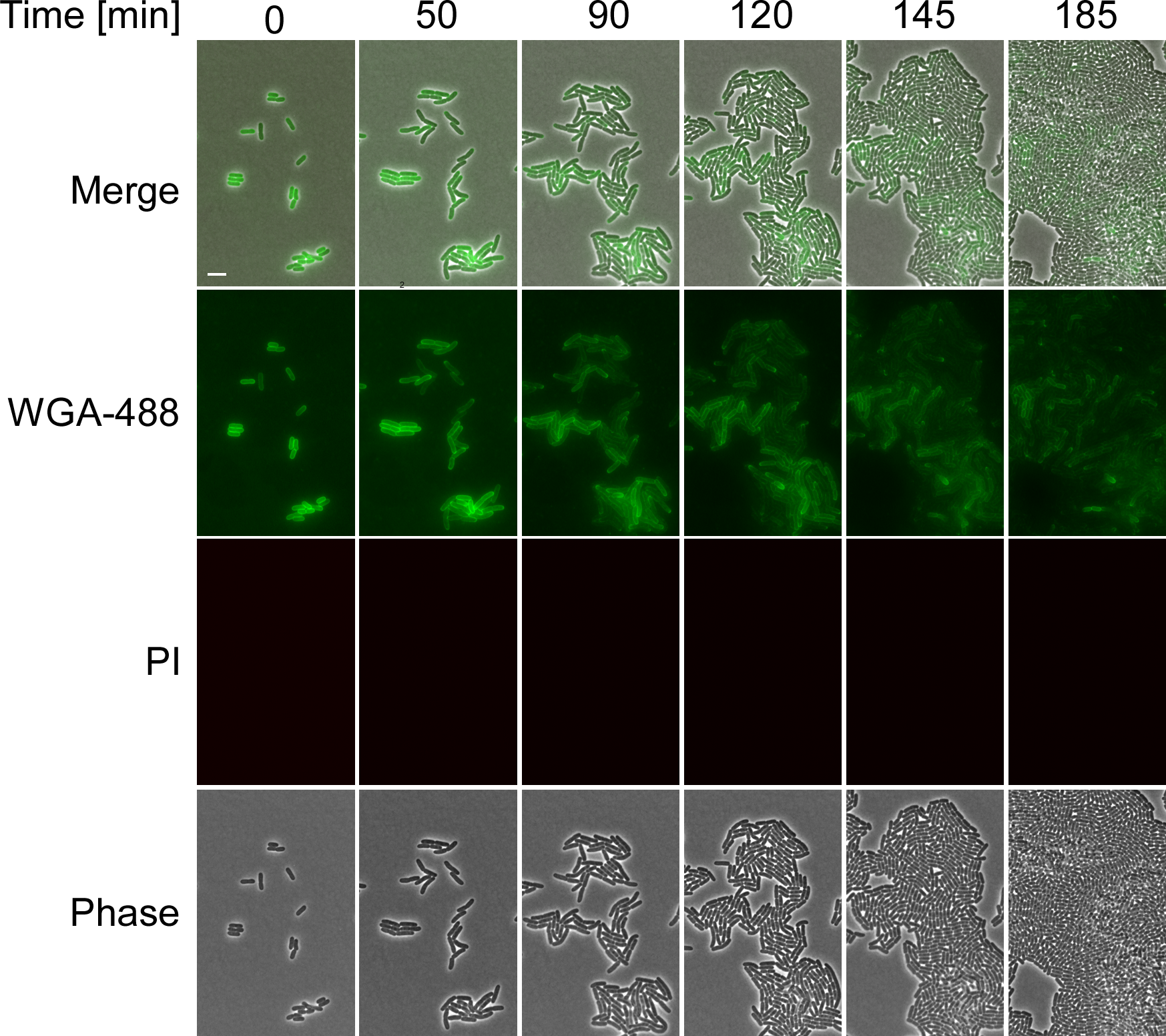

Supplement: FIG S4 [file msystems.00723-22-s0004.tif]
