# Supplementary material for: Multiple T6SSs, Mobile Auxiliary Modules, and Effectors Revealed in a Systematic Analysis of the Vibrio parahaemolyticus Pan-Genome
Source: mSystems. 2022 Oct 13;7(6):e00723-22. doi: 10.1128/msystems.00723-22 (PMC9765294; doi:10.1128/msystems.00723-22)
Supplement: TABLE S1 [file msystems.00723-22-s0005.docx]

**Table S1. A list of bacterial strains and plasmids used in this work.**

| **Strain name** | **Genotype** | **Comments** | **Source** |
| --- | --- | --- | --- |
| *Vibrio parahaemolyticus* RIMD 2210633 | Wild type | Used for generating deletion strains. | (1) |
| *Vibrio parahaemolyticus* RIMD 2210633 ∆*hcp1* | ∆*vp1393* | RIMD 2210633 derivative containing an in-frame deletion of *vp1393;* used as prey in bacterial competition assays. | (2) |
| VpT6SS1^Surrogate^ | ∆*tdhAS*/∆*vp1388/*∆*vpa1263/ vp1415^AAA^*/∆*vp1133* | RIMD 2210633 derivative containing in-frame deletions of *tdhAS*, *vp1388*, *vpa1263*, and *vp1133*, and substitutions of the codons encoding histidines 563-4 in VP1415 with codons for alanines. Used as a surrogate platform in competition assays and in secretion assays. | This study |
| VpT6SS1^Surrogate/∆^*^hcp1^* | ∆*tdhAS*/∆*vp1388/*∆*vpa1263/ vp1415^AAA^*/∆*vp1133/*∆*vp1393* | VpT6SS1^Surrogate^ derivative containing an in-frame deletion of *vp1393;* used as a T6SS1^-^ surrogate platform in competition assays and in secretion assays. | This study |
| *Vibrio natriegens* ATCC 14048 | Wild type | Used as prey in competition assays. | ATCC collection |
| *Vibrio coralliilyticus* ATCC BAA-450 | Wild type | Used for generating deletion strains. | ATCC collection |
| *Vibrio coralliilyticus* ATCC BAA-450 ∆*hcp1* | ∆*vic_rs16330* | ATCC BAA-450 derivative containing an in-frame deletion of *vic_rs16330*; used as prey in competition assays. | This study |
| *Vibrio campbellii* ATCC 25920 | Wild type | Used for generating deletion strains. | ATCC collection |
| *Vibrio campbellii* ATCC 25920 ∆*hcp1* | ∆*a8140_16775* | ATCC 25920 derivative containing an in-frame deletion of *a8140_16775;* used as prey in competition assays. | This study |
| *Vibrio parahaemolyticus* 12-297/B ∆*hcp1* | ∆*b5c30_rs15290* | 12-297/B derivative containing an in-frame deletion of *b5c30_rs15290;* used as prey in competition assays | (3) |
| *Vibrio vulnificus* CMCP6 | Rifampicin-resistant parental strain | Used as prey in competition assays. | Obtained from Karla Satchell |
| *Aeromonas jandaei* DSM 7311 ∆*tssB* | ∆*bn1126_rs13720* | DSM 7311 derivative containing an in-frame deletion of *bn1126_*rs13720; used as prey in competition assays | (4) |
| *Escherichia coli* DH5α (λ pir) | K-12 derivative laboratory strain containing λ pir | Used for plasmid maintenance and cloning. | Obtained from Eric V. Stabb |
| *Escherichia coli* BL21 (DE3) | Laboratory strain | Used for protein expression and toxicity assays. | Obtained from Kim Orth |
| *Escherichia coli* MG1655 | Laboratory strain | Used for microscopy assays. |  |
| **Plasmid name** | **Description** | **Purpose** | **Source** |
| pBAD^K^/Myc-His | pBR322 ori-containing plasmid harboring a Kan^R^ cassette, *araC*, and an MCS following a P*bad* promoter | Used for arabinose-inducible expression | (5) |
| pDUF4225^18764-Cyto^ | pBAD^K^/Myc-His plasmid containing the CDS of DUF4225^18764^ in frame with the C-terminal Myc-His tag | Used for arabinose-inducible expression of DUF4225^18764^ proteins in *E. coli* | This study |
| pPER5 | pBAD^K^/Myc-His with a PelB signal peptide inserted at the 5' end of the MCS | Used for arabinose-inducible expression of proteins targeted to the periplasm in *E. coli* | (6) |
| pDUF4225^18764-Peri^ | pPER5 plasmid containing the CDS of DUF4225^18764^ in frame with the N-terminal PelB signal peptide and the C-terminal Myc-His tag | Used for arabinose-inducible expression of DUF4225^18764^ protein targeted to the periplasm in *E. coli* | This study |
| pTme1^peri^ | pPER5 plasmid containing the CDS of Tme1 from *V. parahaemolyticus* BB22OP in frame with the N-terminal PelB signal peptide and the C-terminal Myc-His tag | Used for arabinose-inducible expression of Tme1 protein targeted to the periplasm in *E. coli* | (7) |
| pTse1^peri^ | pPER5 plasmid containing the CDS of Tse1 (NP_250535.1) from *P. aeruginosa* PAO1 in frame with the N-terminal PelB signal peptide and the C-terminal Myc-His tag | Used for arabinose-inducible expression of Tse1 protein targeted to the periplasm in *E. coli* | This study |
| pBAD33.1^F^ | pBAD33.1 with a FLAG tag inserted at the 3' end of the MCS | Used for arabinose-inducible expression of proteins | (7) |
| pHcp1b | pBAD33.1^F^ plasmid containing the CDS of Hcp1b (WP_065788327.1) from *V. parahaemolyticus* strain CFSAN018764 in frame with the C-terminal FLAG tag of the plasmid | Used for arabinose-inducible expression of Hcp1b in *V. parahaemolyticus* | This study |
| pModule | pBAD33.1^F^ containing the Hcp1b module (Hcp1b, DUF4225^18764^, and Imm4225^18764^) from *V. parahaemolyticus* strain CFSAN018764; Imm4225^18764^ is cloned in-frame with the C-terminal Flag tag of the plasmid | Used for arabinose-inducible expression of the Hcp1b module in *V. parahaemolyticus* | This study |
| pPoNe | pBAD33.1^F^ containing the *V. parahaemolyticus* 12-297/B VgrG1b auxiliary module with the effector and immunity pair PoNe/i*^Vp^* ^12-297/B^ (*b5c30_rs14470-60*); PoNi*^Vp^* ^12-297/B^ is cloned in-frame with the C-terminal FLAG tag of the plasmid | Used for arabinose-inducible expression of the PoNe/i*^Vp^* ^12-297/B^ effector and immunity pair-containing VgrG1b module in *V. parahaemolyticus* | This study |
| pImm4225^18764^ | pBAD33.1^F^ plasmid containing the CDS of Imm4225^18764^ in frame with the C-terminal FLAG tag of the plasmid | Used for arabinose-inducible expression of Imm4225^18764^ in *E. coli* | This study |
| pDM4 | a *Cm^R^* and *oriV_R6K_*-containing suicide vector | Used to generate deletions and substitutions in *Vibrio* genomes | (8) |
| pDM4:*hns* | pDM4 containing 1 kb upstream and 1 kb downstream of *vp1133* in its MCS | Used to delete *hns* in *V. parahaemolyticus* RIMD 2210633 | (9) |
| pDM4:*hcp1* | pDM4 containing 1 kb downstream and 1 kb upstream of *vp1393* in its MCS | Used to delete *hcp1* in *V. parahaemolyticus* RIMD 2210633 | (5) |
| pDM4:*vp1388* | pDM4 containing 1 kb upstream and 1 kb downstream of *vp1388* in its MCS | Used to delete *vp1388* in *V. parahaemolyticus* RIMD 2210633 | (10) |
| pDM4:*vpa1263* | pDM4 containing 1 kb upstream and 1 kb downstream of *vpa1263* in its MCS | Used to delete *vpa1263* in *V. parahaemolyticus* RIMD 2210633 | (10) |
| pDM4:*vp1415*^AAA^ | pDM4 containing ~2.2 kb region encompassing 1.1 kb upstream and 1.1 kb downstream of the codons encoding histidines 563-4 in *V. parahaemolyticus* RIMD 2210633 *vp1415* in which these two codons were substituted to encode alanines | Used to substitute VP1415 histidines 563-4 for alanine in the *V. parahaemolyticus* RIMD 2210633 genome | (11) |
| pCLTR | *E. coli*-yeast-*Vibrio* shuttle vector; mobilizable; it contains Strep^R^, Spec^R^ and Cm^R^ | Used to express proteins in *V. natriegens* | (11) |
| pImm | pCLTR containing the region encompassing *araC* to the rrnT1 terminator, amplified from the pImm4225^18764^ plasmid | Used for arabinose-inducible expression of Imm4225^18764^ in *V. natriegens* | This study |

**References**

1. Makino K, Oshima K, Kurokawa K, Yokoyama K, Uda T, Tagomori K, Iijima Y, Najima M, Nakano M, Yamashita A, Kubota Y, Kimura S, Yasunaga T, Honda T, Shinagawa H, Hattori M, Iida T. 2003. Genome sequence of Vibrio parahaemolyticus: a pathogenic mechanism distinct from that of V cholerae. Lancet 361:743–749.

2. Dar Y, Jana B, Bosis E, Salomon D. 2022. A binary effector module secreted by a type VI secretion system. EMBO Rep 23:e53981.

3. Jana B, Fridman CM, Bosis E, Salomon D. 2019. A modular effector with a DNase domain and a marker for T6SS substrates. Nat Commun 10:3595.

4. Jana B, Keppel K, Salomon D. 2021. Engineering a customizable antibacterial T6SS‐based platform in Vibrio natriegens. EMBO Rep 22:e53681.

5. Salomon D, Gonzalez H, Updegraff BL, Orth K. 2013. Vibrio parahaemolyticus Type VI secretion system 1 Is activated in marine conditions to target bacteria, and is differentially regulated from system 2. PLoS One 8:e61086.

6. Dar Y, Salomon D, Bosis E. 2018. The antibacterial and anti-eukaryotic Type VI secretion system MIX-effector repertoire in Vibrionaceae. Mar Drugs 16:433.

7. Fridman CM, Keppel K, Gerlic M, Bosis E, Salomon D. 2020. A comparative genomics methodology reveals a widespread family of membrane-disrupting T6SS effectors. Nat Commun 11:1085.

8. O’Toole R, Milton DL, Wolf-Watz H. 1996. Chemotactic motility is required for invasion of the host by the fish pathogen Vibrio anguillarum. Mol Microbiol 19:625–637.

9. Salomon D, Klimko JA, Orth K. 2014. H-NS regulates the Vibrio parahaemolyticus type VI secretion system 1. Microbiol (United Kingdom) 160:1867–1873.

10. Salomon D, Kinch LN, Trudgian DC, Guo X, Klimko JA, Grishin N V., Mirzaei H, Orth K. 2014. Marker for type VI secretion system effectors. Proc Natl Acad Sci 111:9271–9276.

11. Jana B, Keppel K, Salomon D. 2021. Engineering a customizable antibacterial T6SS-based platform in Vibrio natriegens. EMBO Rep 22:e53681.
